# Novel Cellulose Triacetate (CTA)/Cellulose Diacetate (CDA) Blend Membranes Enhanced by Amine Functionalized ZIF-8 for CO_2_ Separation

**DOI:** 10.3390/polym13172946

**Published:** 2021-08-31

**Authors:** Ayesha Raza, Susilo Japip, Can Zeng Liang, Sarah Farrukh, Arshad Hussain, Tai-Shung Chung

**Affiliations:** 1 Department of Chemical and Biomolecular Engineering, National University of Singapore, Singapore 117585, Singapore; ayesha.raza@scme.nust.edu.pk (A.R.); a0096057@u.nus.edu (S.J.); chelian@nus.edu.sg (C.Z.L.); 2 Department of Chemical and Materials Engineering, National University of Sciences and Technology, Islamabad 44000, Pakistan; Sarah.farrukh@scme.nust.edu.pk; 3 Department of Chemical and Energy Engineering, Pak-Austria University, Haripur 22621, Pakistan; arshad.hussain@fcm3.paf-iast.edu.pk; 4 Department of Chemical Engineering, National Taiwan University of Science and Technology, Taipei 1061, Taiwan

**Keywords:** zeolitic imidazolate frameworks, polymer blend, mixed matrix membrane, plasticization resistance, CO_2_ separation

## Abstract

Currently, cellulose acetate (CA) membranes dominate membrane-based CO_2_ separation for natural gas purification due to their economical and green nature. However, their lower CO_2_ permeability and ease of plasticization are the drawbacks. To overcome these weaknesses, we have developed high-performance mixed matrix membranes (MMMs) consisting of cellulose triacetate (CTA), cellulose diacetate (CDA), and amine functionalized zeolitic imidazolate frameworks (NH_2_-ZIF-8) for CO_2_ separation. The NH_2_-ZIF-8 was chosen as a filler because (1) its pore size is between the kinetic diameters of CO_2_ and CH_4_ and (2) the NH_2_ groups attached on the surface of NH_2_-ZIF-8 have good affinity with CO_2_ molecules. The incorporation of NH_2_-ZIF-8 in the CTA/CDA blend matrix improved both the gas separation performance and plasticization resistance. The optimized membrane containing 15 wt.% of NH_2_-ZIF-8 had a CO_2_ permeability of 11.33 Barrer at 35 °C under the trans-membrane pressure of 5 bar. This is 2-fold higher than the pristine membrane, while showing a superior CO_2_/CH_4_ selectivity of 33. In addition, the former had 106% higher CO_2_ plasticization resistance of up to about 21 bar and an impressive mixed gas CO_2_/CH_4_ selectivity of about 40. Therefore, the newly fabricated MMMs based on the CTA/CDA blend may have great potential for CO_2_ separation in the natural gas industry.

## 1. Introduction

Natural gas is an attractive and relatively green energy source as compared to coal, mainly because of its lower carbon footprint [1,2,3]. However, depending on the geological location, raw natural gas varies substantially in composition and may contain 50–90 mole % methane together with other undesirable components such as H_2_O, CO_2_, H_2_S, N_2_, C_2_H_6_, C_3_H_8_, and toluene. The presence of CO_2_ and H_2_S can cause pipeline corrosion and reduce the caloric value of the natural gas [1,4,5]. Therefore, the demand of high-purity natural gas is increasing by the energy producers to enhance its calorific and economic values [5]. To purify raw natural gas, membrane technology has emerged as an alternative process due to its environmental and economic benefits [1,5]. In particular, polymeric membranes have led to the commercialization of membrane-based gas separations for various applications including CO_2_ separation from natural gas [6,7,8]. To make the membrane-based gas separation more competitive over conventional separation technologies such as amine absorption and cryogenic distillation, membranes with a higher selectivity and permeability are required [9,10,11,12,13]. Nevertheless, the design of high-performance polymeric membranes is challenging mainly due to (1) the tradeoff relationship between permeability and selectivity of the polymeric materials [14] and (2) the plasticization phenomenon because the highly condensable gases like CO_2_ tend to drastically reduce membrane selectivity [15,16,17].

Tremendous efforts have been made to overcome these constraints and to design novel membrane materials with high separation performance in terms of both gas selectivity and permeability [9,10,11,18]. Among these efforts, polymer blends and mixed matrix membranes (MMMs) are the popular approaches because they are simple and effective [19,20]. Particularly, MMMs, which incorporate inorganic particles in the continuous phase of the polymer matrix, have gained growing interests [9,20,21,22]. In MMMs, the polymeric matrix offers low cost and easy processability as the major benefits, while inorganic components exhibit high permeability, selectivity, and good thermal stability. MMMs possess impressive gas separation performance due to the combined benefits of both polymeric and inorganic materials. Inorganic particles incorporated in MMMs as the dispersed phase can be classified into two major categories: (1) non-porous/impermeable fillers such as silica and TiO_2_ and (2) porous/permeable fillers such as carbon nanotubes, zeolites, and metal organic frameworks (MOFs) [20,22].

Currently, MOFs, especially zeolitic imidazolate frameworks (ZIFs), have been receiving significant attention as potential porous fillers used in MMMs owing to their molecular sieving properties, good stability, and compatibility with the polymer matrices [23]. One of the most extensively studied ZIFs is ZIF-8, which is constructed by a sodalite crystal structure. It possesses a pore cavity of 1.16 nm that is reachable through a small pore aperture of 0.34 nm [23]. Among various ZIFs, ZIF-8 has shown a remarkable CO_2_ separation performance [21,24,25] mainly because the aperture size of ZIF-8 is between the kinetic diameters of CO_2_ and CH_4_ (0.33 nm and 0.38 nm, respectively), thus enhancing the CO_2_ diffusivity and permeability [26]. Nevertheless, pore blockage, rigidification of the polymeric chains, and particles agglomeration are the major challenges during the fabrication of MMMs [9,27,28].

Aside from MMMs, polymer blends [19,29,30,31,32,33,34] are one of the practical methods to tackle the aforementioned tradeoff relationship existing in polymeric membranes. Not only can they combine the desirable properties of different materials into the new blend with targeted performance, but they also minimize the deficiencies of the individual components. Apart from their advantages, the major challenge of polymer blends is their miscibility at molecular level [19]. For example, Sanaeepur et al. studied the gas separation performance of CA/Pebax blend membranes [32]. The membrane blended with 8 wt.% Pebax showed 29% and 59% increases in CO_2_ permeability and CO_2_/N_2_ selectivity, respectively. Recently, Akbarzadeh et al. fabricated green blend membranes of thiazole-based polyamine (PM-4) and CA for CO_2_ separation [33]. Their optimal membrane displayed an impressive CO_2_ permeability of up to 3000 Barrer with a CO_2_/CH_4_ selectivity of around 34 obtained at a feed pressure of 3 bar and 35 °C. Lin et al. studied the effects of membrane thickness and CA blend composition of submicron films [34]. They found that the 75/25 (*w*/*w*) cellulose triacetate (CTA)/cellulose diacetate (CDA) blend film with a thickness of 1 µm had a CO_2_ permeability of 14 Barrer, which was not only about 100% higher than the same blend membrane with a thickness of 20 µm (i.e., 14 vs. 7.1 Barrer), but also about 250% higher than the pristine CDA membrane (i.e., 14 vs. 3.9 Barrer). Their findings inspired us to employ CTA/CDA blends as the matrix materials for the fabrication of MMMs for CO_2_/CH_4_ separation.

Therefore, the objectives of this work are to (1) synergistically combine the strengths of MMMs and polymer blends and (2) design a novel membrane material for CO_2_ separation from natural gas. To our best knowledge, the noteworthy combination of CTA/CDA–amine functionalized ZIF-8 polymer blend MMMs has not yet been explored. This would be the first study on the fabrication of CTA/CDA-NH_2_-ZIF-8 blend MMMs and investigation of their CO_2_/CH_4_ separation performance as a function of NH_2_-ZIF-8 loading. CTA and CDA blends were chosen as the matrix material because they have similar chemical structures, good separation performance, and a cheap and environmentally friendly nature. NH_2_-ZIF-8 was used as a filler because its pore size is between the kinetic diameters of the separating gases (CO_2_ and CH_4_). Moreover, the NH_2_ group attached on the surface of ZIF-8 has good affinity with condensable gases like CO_2_. This study may provide useful insights to design next-generation CA membranes for the purification of natural gas.

## 2. Experiments and Methods

### 2.1. Materials

**Figure 1:**
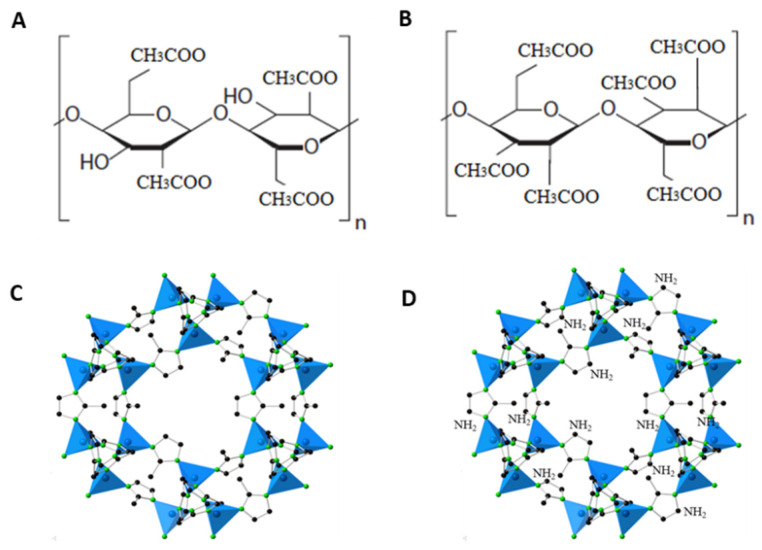
Chemical structure of (**A**) cellulose diacetate (CDA), (**B**) cellulose triacetate (CTA), (**C**) ZIF-8, and (**D**) NH_2_-ZIF-8, Reprinted with permission from ref. [37]. Copyright 2013 Springer Nature.

Cellulose triacetate (CTA) with a degree of substitution (DS) of 2.87 and cellulose diacetate (CDA) with a DS of 2.45 were provided by Eastman Chemical Company (Kingsport, TN, USA), while N-methyl-2-pyrrolidone (NMP, >99.5%) was bought from Merck (Darmstadt, Germany). Zinc nitrate (Zn(NO_3_)_2_·6H_2_O), 2-methylimidazole, and ammonium hydroxide (NH_4_OH) were purchased from Science Centre, Pakistan (Islamabad, Pakistan). Gas cylinders of CO_2_ (purity ≥ 99.95%) and CH_4_ (purity ≥ 99.95%) were supplied by Air Liquide Singapore Pte. LtdSingapore The chemical structures of CTA and CDA are shown in Figure 1A,B.

### 2.2. ZIF-8 Synthesis

A solvo-chemical method was employed to synthesize ZIF-8 nanoparticles as reported by Pan et al. [35]. In this process, Zn(NO_3_)_2_·6H_2_O (1.17 g) and 2-methylimidazole (22.70 g) were dissolved separately in deionized (DI) water. The prepared solutions were stirred for a few minutes prior to the mixing and were continuously stirred at room temperature for another 12 h. The resultant milky solution was centrifuged, followed by washing with DI water. Afterwards, the washed product was dried in an oven at 65 °C overnight. 

### 2.3. Amine Modification of ZIF-8

Amine functionalization of ZIF-8 nanoparticles was carried out following the method reported by Nordin et al. [36], with some modifications. Briefly, NH_4_OH (28 mL) and H_2_O (10 mL) were slowly added to ZIF-8 (1 g) under constant stirring, followed by overnight sonication at 80 °C. The resulting mixture was centrifuged and washed multiple times with distilled water to remove any impurities before being dried in a vacuum oven at 70 °C for 12 h. The chemical structures of ZIF-8 and NH_2_-ZIF-8 are presented in Figure 1C,D respectively [37].

### 2.4. Membrane Fabrication

Solution casting and solvent evaporation techniques were used to fabricate dense membranes. CDA and CTA powders were dried in a vacuum oven at 120 °C overnight to remove moisture. The blend polymer consisting of 80/20 (wt.%) CTA/CDA was dissolved in NMP followed by vigorous stirring at 40 °C overnight until the solution became homogeneous. Separately, the NH_2_-ZIF-8 and NMP suspension was sonicated for 2 h prior to mixing with the pre-prepared polymer blend solution. After mixing, the solution was stirred for 30 min and then cast on a clean glass plate by a casting blade, followed by solvent evaporation in a conventional oven at 100 °C overnight. The dried films were peeled off from the glass plate and dried in a vacuum oven at 120 °C for at least 24 h. The loading of NH_2_-ZIF-8 was calculated using Equation (1) and varied from 0 to 15 wt.% based on the polymer weight. For example, a 5 wt.% NH_2_-ZIF-8 MMM consists of 95 wt.% the blend polymer (i.e., 76 wt.% CTA and 19 wt.% CDA) and 5 wt.% NH_2_-ZIF-8.

(1) $$Particle\;loading\;(\%)=\frac{weight\;of\;the\;particles}{weight\;of\;the\;particles+weight\;of\;the\;polymer}\times 100$$

### 2.5. Characterizations

The crystal structure of NH_2_-ZIF-8 nanoparticles and the corresponding MMMs were analyzed by a Bruker wide-angle X-ray diffractometer (Bruker D8 advanced diffractometer, Bruker, Tokyo, Japan) using Cu-Kα as a radiation source at a wavelength of 1.54 Ǻ. The morphologies of the samples were examined by a field emission scanning electron microscope (FESEM, JEOL, JSM-6700LV, Tokyo, Japan). Prior to the inspection, the membranes were frozen and fractured in liquid nitrogen followed by platinum coating using a platinum (Pt) sputter coater (JEOL JFC-1300, Peabody, MA, USA). NH_2_-ZIF-8 nanoparticles were directly glued on the surface of the sample holder before being coated. Chemical functionalities and interactions between polymers and nanofillers were confirmed by Fourier-transform infrared spectroscopy (FTIR, spectrum 100, Perkin Elmer, Beaconsfield, England, UK). The measurements were conducted between the wave numbers ranging from 4000 to 600 cm^−1^. Thermal stabilities of pure NH_2_-ZIF-8 nanoparticles and MMMs were examined by a Shimadzu Thermal Analyzer (DTG-60AH/TA-60WS/FC-60A, Shimadzu corporation, Tokyo, Japan). All samples were heated at a heating rate of 10 °C/min from room temperature to 800 °C under air atmosphere. 

### 2.6. Gas Permeation Measurements

#### 2.6.1. Pure Gas Tests

A constant volume variable pressure method was used to record the pure gas permeation properties. Detailed experimental setup and procedures can be found elsewhere [38]. All the membranes were tested at 35 °C under the trans-membrane pressure of 5 bar. For each membrane, at least three samples were tested and the average results were reported. The gas permeability was calculated based on the rate change of the downstream pressure increase (*dp*/*dt*) at steady state by using Equation (2)

(2) $$P=\frac{273\times 10^{10}}{760}\frac{VL}{AT(p_{2}\times 76/14.7)}\frac{dp}{dt}$$

where *P* is the membrane permeability in Barrer (1 Barrer = 1 × 10^−10^ cm^3^(STP) cm/(cm^2^ s cmHg), *L* is the membrane thickness (cm), *V* represents the volume of the downstream chamber (cm^3^), *A* is the effective membrane area (cm^2^), *T* signifies the operating temperature (K), and the upstream pressure is represented by *p*_2_ (psia).

The ideal selectivity (*α_A/B_*) of two gases (A and B) was calculated according to Equation (3)

(3) $$\alpha_{A/B}=P_{A}/P_{B}=(S_{A}/S_{B})\times (D_{A}/D_{B})$$

where *P_A_* and *P_B_* represent the permeability (Barrer) of gases A and B, respectively. *S* and *D* denote the solubility and diffusion coefficients of the gas, respectively. *S_A_/S_B_* and *D_A_/D_B_* are the solubility selectivity and diffusion selectivity of the gas pair, respectively.

The sorption behaviors of the blend membrane and the optimized MMM were evaluated using a XEMIS microbalance. Detailed experimental procedures can be found elsewhere [39]. Equation (4) was used to estimate the solubility coefficient (S, m^3^(STP)/cm^3^cmHg) of the adsorbed gas inside the membrane at 5 bar, while the diffusivity coefficient (D, 10^−8^ cm^2^/s) was calculated based upon Equation (5).

(4) $$S=\frac{C}{p}$$

(5) P=S×D

where *C* represents the total adsorbed gas concentration (cm^3^(STP)/cm^3^) and *p* is the feed pressure (cmHg).

#### 2.6.2. Mixed Gas Tests

Mixed gas tests were carried out at 10 bar and 35 °C using a binary feed mixture of CO_2_/CH_4_ (50/50 *v*/*v*). The detailed experimental description can be found elsewhere [40]. The permeability for each gas was calculated using Equation (6)

(6) $$P_{i}=\frac{273\times 10^{10}}{760}\frac{y_{i}VL}{AT(76/14.7)(x_{i}p_{2})}\frac{dp_{1}}{dt}$$

where *P_i_* is the permeability of gas *i*, *p*_2_ represents the upstream feed gas pressure (psia), *p*_1_ is the downstream pressure (psia) of the permeate gas, *x_i_* is the molar fraction of gas *i* in the feed gas stream and *y_i_* is the molar fraction of gas *i* in the permeate, *L* is the membrane thickness (cm), and *V* signifies the volume of the downstream chamber (cm^3^). 

## 3. Results and Discussion

### 3.1. Characterizations

**Figure 2:**
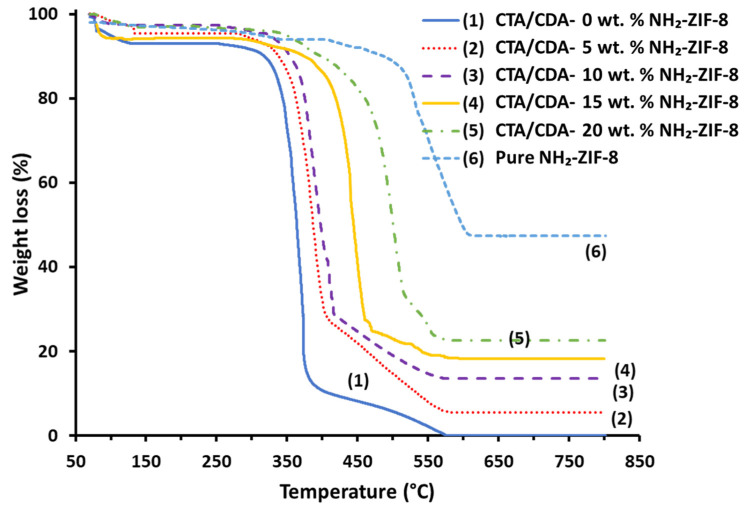
TGA of pure NH_2_-ZIF-8 and CTA/CDA-NH_2_-ZIF-8 MMMs with different NH_2_-ZIF-8 loadings.

Figure 2 shows the weight loss profiles of the pure NH_2_-ZIF-8 nanoparticles, CTA/CDA blend membrane, and fabricated MMMs as a function of temperature. The NH_2_-ZIF-8 nanoparticles exhibited a gradual weight loss of around 9 wt.% from 30 to 450 °C owing to the removal of guest molecules trapped in the nanocrystals during synthesis and post treatment steps, followed by a steep decrease from 450 to 600 °C due to the framework decomposition. The findings are in good agreement with the reported thermal behavior of ZIF-8 [41,42]. The TGA thermogram of the pure CTA/CDA blend membrane revealed three weight-loss steps which are consistent with the literature [43,44,45]. The initial weight loss from 30 to 120 °C corresponded to the removal of volatile matters and moisture adsorbed by the membrane due to the hygroscopic nature of CA. The second major weight loss in the range of 310–400 °C symbolized the thermal degradation of the polymer followed by the third step due to the carbonization of degraded products to ash. Thermograms of MMMs also exhibited these three steps of weight loss and demonstrate good thermal stability up to 310 °C. Table 1 shows a significant improvement in decomposition temperature (Td) with an increase in NH_2_-ZIF-8 loading. The improvement in Td arose from (1) good compatibility and strong interaction between the filler and the polymer matrix and (2) inherent thermal characteristics of NH_2_-ZIF-8 nanoparticles. Thus, the incorporation of NH_2_-ZIF-8 into the CTA/CDA blend membrane hindered the chain movement and raised the energy requirement to decompose its polymer structure. A similar observation has been widely reported in the literature [46,47,48].

**Figure 3:**
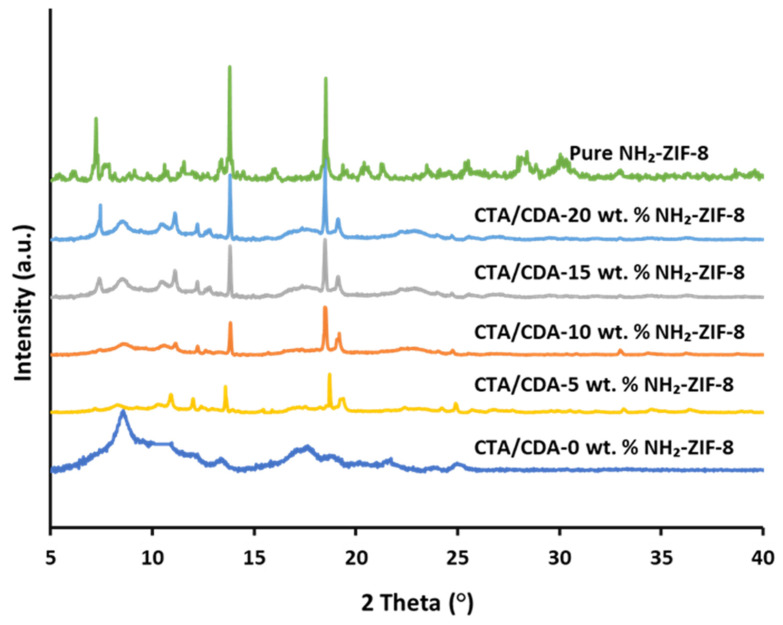
XRD analyses of pure NH_2_-ZIF-8 and CTA/CDA-NH_2_-ZIF-8 MMMs with different NH_2_-ZIF-8 loadings.

Figure 3 displays the XRD patterns of the pure NH_2_-ZIF-8 nanoparticles, CTA/CDA blend membrane, and their MMMs as a function of NH_2_-ZIF-8 loading. The two main peaks at 2θ values of 8° and 17° confirm the semi-crystalline nature of the CTA/CDA blend membrane [49]. The XRD pattern of the NH_2_-ZIF-8 nanocrystals was also in good agreement with the literature [50,51]. All the diffraction patterns of CTA/CDA-NH_2_-ZIF-8 MMMs possessed the characteristic peaks of both NH_2_-ZIF-8 and CTA/CDA moieties, signifying the preservation of their crystalline structures in the membranes. In addition, the intensity of the characteristic peaks of NH_2_-ZIF-8 improved with an increase in its loading in MMMs.

**Figure 4:**
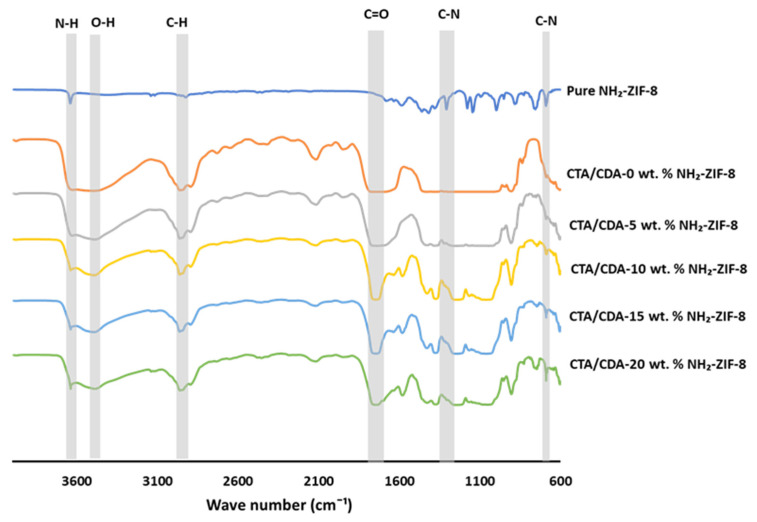
FTIR analyses of pure NH_2_-ZIF-8 and CTA/CDA-NH_2_-ZIF-8 MMMs with different NH_2_-ZIF-8 loadings.

FTIR spectra of NH_2_-ZIF-8 nanoparticles and all fabricated membranes are compared in Figure 4. The CTA/CDA spectrum was characterized by major peaks at 3483, 2950, and 1750 cm^−1^ corresponding to O-H, C-H, and C=O groups, respectively [52]. In contrast, the FTIR spectrum of NH_2_-ZIF-8 nanoparticles was in good agreement with the reported literature [52,53]. The amine functionalization of ZIF-8 was also confirmed by the absorption peaks at ~1309 cm^−1^ and 690 cm^−1^ due to the NH_2_ bonding on ZIF-8 molecules [54]. In summary, the FTIR spectra reconfirmed the existence of CTA/CDA and NH_2_-ZIF-8 nanoparticles in all fabricated MMMs.

**Figure 5:**
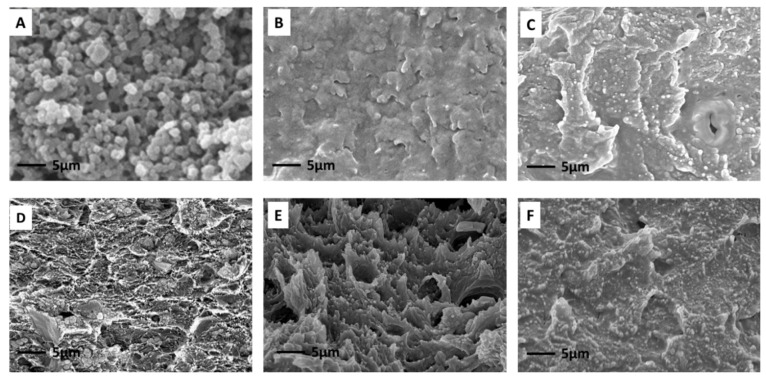
FESEM cross-section images of (**A**) pure NH_2_-ZIF-8, (**B**) CTA/CDA-0 wt.% NH_2_-ZIF-8, (**C**) CTA/CA-5 wt.% NH_2_-ZIF-8, (**D**) CTA/CDA-10 wt.% NH_2_-ZIF-8, (**E**) CTA/CDA-15 wt.% NH_2_-ZIF-8, and (**F**) CTA/CDA-20 wt.% NH_2_-ZIF-8.

**Figure 6:**
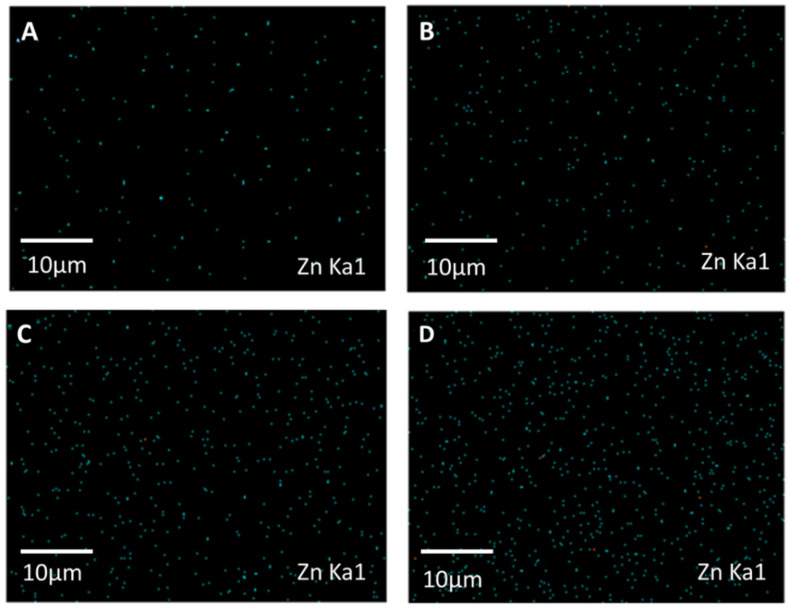
EDX analyses of (**A**) CTA/CDA-5 wt.% NH_2_-ZIF-8, (**B**) CTA/CDA-10 wt.% NH_2_-ZIF-8, (**C**) CTA/CDA-15 wt.% NH_2_-ZIF-8, and (**D**) CTA/CDA-20 wt.% NH_2_-ZIF-8.

Figure 5 displays the FESEM images of the pure NH_2_-ZIF-8 nanoparticles, CTA/CDA blend membrane, and their MMMs as a function of NH_2_-ZIF-8 loading. The NH_2_-ZIF-8 nanoparticles had a size between 50 and 80 nm. Meanwhile, they were well dispersed in the CTA/CDA matrix with no clear evidence of interfacial gaps and phase separation. This signifies the good compatibility between the NH_2_-ZIF-8 nanoparticles and CTA/CDA matrix because of (1) the small size of NH_2_-ZIF-8 nanoparticles and (2) the formation of hydrogen bonds between the −NH_2_ groups of NH_2_-ZIF-8 nanoparticles and the −OH groups of the polymer matrix [5]. The uniform dispersion of NH_2_-ZIF-8 nanoparticles was also confirmed by the energy-dispersive X-ray spectroscopy (EDX). Figure 6 shows the mapping of Zn particles in the MMMs with variable loadings. Since the polymer matrix did not contain any Zn element, the uniform dispersion of Zn element verified the uniform dispersion of NH_2_-ZIF-8 nanocrystals in the polymer matrix.

### 3.2. Gas Transport Properties

**Figure 7:**
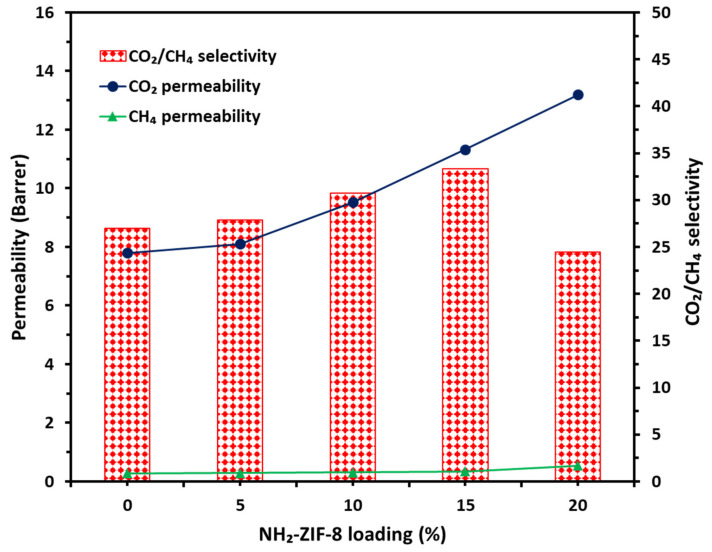
Separation performance of fabricated membranes as a function of NH_2_-ZIF-8 loading.

Pure gas separation performances of the CTA/CDA blend membrane and CTA/CDA-NH_2_-ZIF-8 MMMs are shown in Figure 7. Table 1 tabulates the detailed results. Both CO_2_ and CH_4_ permeabilities exhibited noticeable increases as a function of NH_2_-ZIF-8 loading. However, the CO_2_/CH_4_ selectivity exhibited an up and down trend. A similar trend was observed for polyimide-ZIF-8 MMMs in the literature [55]. Generally, the incorporation of nanofillers in the polymer matrix disrupts the packing of polymeric chains, which may result in additional free volume and diffusion paths for gas transport [56]. Therefore, the CTA/CDA-15 wt.% NH_2_-ZIF-8 membrane had a 50% increase in CO_2_ permeability from 7.56 to 11.33 Barrer and a 23% increase in CO_2_/CH_4_ selectivity from 27.0 to 33.3 compared to the neat CTA/CDA blend membrane. The higher selectivity may have resulted from the enhanced molecular sieving capability provided by NH_2_-ZIF-8 as its aperture size (3.4 Å) is between the kinetic diameters of CO_2_ (3.3 Å) and CH_4_ (3.8 Å) [26], while the larger permeability may have arisen from strong interaction among CO_2_, imidazole linkers, and -NH_2_ groups of NH_2_-ZIF-8 that facilitated gas transport across the membrane [57]. However, a further increment in NH_2_-ZIF-8 loading to 20 wt.% resulted in a lower CO_2_/CH_4_ selectivity of 24.4 but a higher CO_2_ permeability of 13.2 Barrer. This is because a higher loading may generate nonselective voids. As a consequence, the CTA/CDA-15 wt.% NH_2_-ZIF-8 blend MMM was selected for subsequent investigations.

#### 3.2.1. Sorption Behavior of CO_2_ and CH_4_

**Figure 8:**
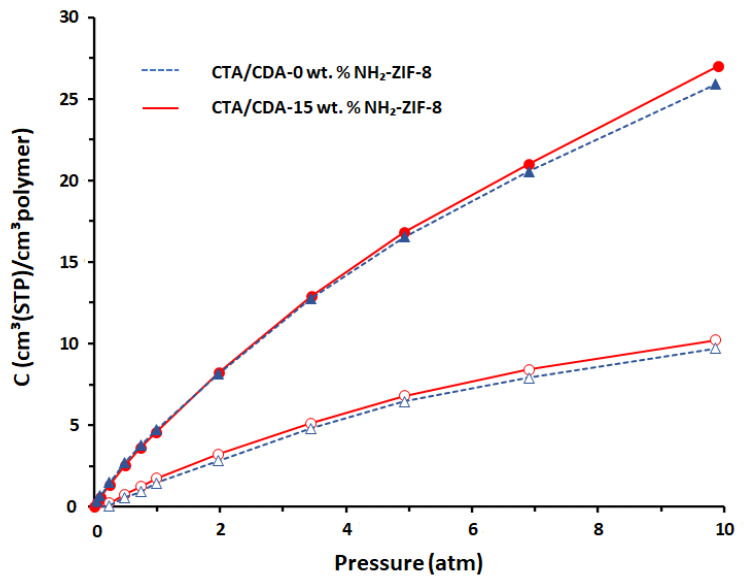
Sorption isotherms of CO_2_ (closed symbols) and CH_4_ (open symbols) in the pristine CTA/CDA-0 wt.% NH_2_-ZIF-8 (solid blue line) and CTA/CDA-15 wt.% NH_2_-ZIF-8 MMM (solid red line) as a function.

Figure 8 presents the dual-mode Langmuir–Henry sorption behavior for both CO_2_ and CH_4_ in the pristine polymer blend and the optimized MMM. Both membranes showed higher CO_2_ adsorption than CH_4_ because of the inherently higher sorption affinity between the CTA/CDA polymer and CO_2_ [58]. Table 2 summarizes their permeability, solubility, diffusivity coefficients, and selectivity. As expected, the CO_2_ diffusivity and solubility coefficients showed 44% and 4% increases, respectively, when 15 wt.% NH_2_-ZIF-8 was incorporated into the CTA/CDA blend matrix. Consequently, the overall improvement in gas separation performance came from two factors; namely, the additional diffusive pathways and free volume provided by NH_2_-ZIF-8. However, comparing the percentages of their increments, one can easily conclude that the former played a more important role than the latter to enhance the molecular sieving capability of the CTA/CDA blend membrane for CO_2_/CH_4_ separation, as observed in the literature [59]. 

#### 3.2.2. Plasticization Behavior and Mixed Gas Tests

**Figure 9:**
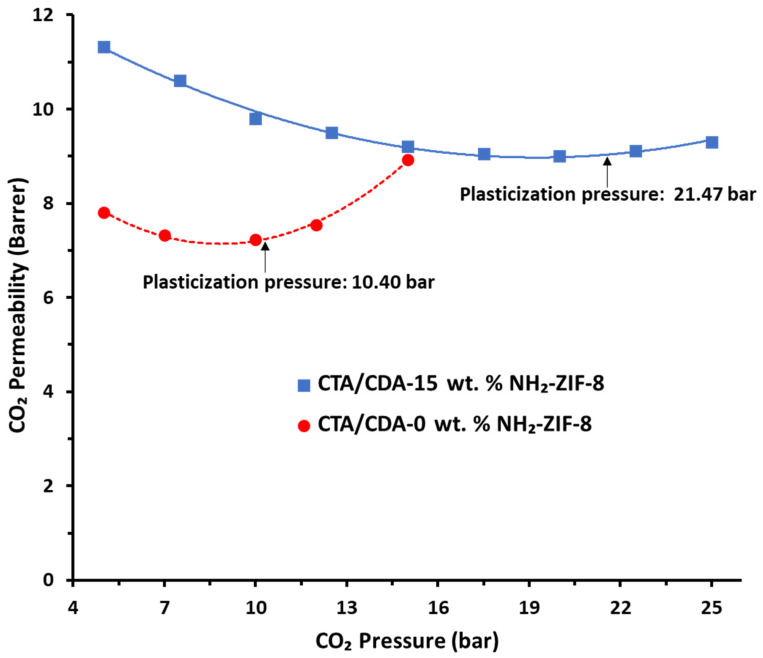
CO_2_ plasticization behavior of the pristine CTA/CDA-0 wt.% NH_2_-ZIF-8 blend membrane and CTA/CDA-15 wt.% NH_2_-ZIF-8 blend MMM.

To investigate the CO_2_-induced plasticization phenomenon, the testing pressure of CO_2_ was intermittently ramped from 5 to 25 bar at 35 °C. Figure 9 shows the CO_2_-induced plasticization behavior of the pristine CTA/CDA blend membrane and the CTA/CDA-15 wt.% NH_2_-ZIF-8 MMM. The former had a plasticization pressure of around 10.4 bar, while the latter showed a plasticization pressure of about 21.47 bar. The notable improvement in CO_2_-induced plasticization pressure may have arisen from good compatibility and chain interactions between the polymer matrix and NH_2_-ZIF-8 molecules. The results are in good agreement with the literature [60].

Table 3 compares the gas separation performance of the pristine CTA/CDA blend membrane and the optimized MMM under pure and mixed gas tests where a binary mixture of CO_2_/CH_4_ (50/50, *v*/*v*) was used as the mixed gas feed. Consistent with the literature, the mixed gas tests showed lower permeabilities for both CO_2_ and CH_4_ gases than the pure gas ones owing to the competitive diffusion and sorption between CO_2_ and CH_4_ molecules [61,62]. Since the percentage of permeability drop for CH_4_ in the former was higher than that in the latter, this results in a higher CO_2_/CH_4_ selectivity in the mixed gas tests.

### 3.3. Benchmark with the Literature

Table 4 benchmarks the pure-gas separation performance of the newly developed membranes with other CA membranes reported in the literature for CO_2_ and CH_4_ separation. The CTA/CDA-15 wt.% NH_2_-ZIF-8 membrane showed higher separation performance because its polymer matrix was made of a CTA/CDA blend that had inherently high gas separation performance and (2) it had 15 wt.% NH_2_-ZIF-8 nanoparticles to enhance its gas diffusivity and molecular sieving characteristics.

## 4. Conclusions

We fabricated high-performance CTA/CDA-NH_2_-ZIF-8 MMMs for CO_2_/CH_4_ separation. A solvo-chemical method was employed to synthesize NH_2_-ZIF-8 nanocrystals with a particle size of 50–80 nm. Gas separation performance of the fabricated membranes have been investigated as a function of NH_2_-ZIF-8 loading. The following conclusions can be drawn:

1. There is a linear relationship between permeability and NH_2_-ZIF-8 loading. However, the relationship changes to a ˄-shape between CO_2_/CH_4_ selectivity and NH_2_-ZIF-8 loading due to void formation. Thus, there is an optimum loading to fabricate CTA/CDA based MMMs with high gas separation performance.
2. The optimized MMM contained 15 wt.% of NH_2_-ZIF-8 nanoparticles and had a CO_2_ permeability of 11.33 Barrer which was 50% higher than the pristine CTA/CDA membrane. In addition, the former had a superior CO_2_/CH_4_ selectivity of 33.33 to the latter of 27 under pure gas tests.
3. The enhanced molecular sieving capability and additional free volume provided by NH_2_-ZIF-8 nanoparticles improved the CO_2_ diffusivity and solubility coefficients by 44% and 4%, respectively, under pure gas tests when 15 wt.% NH_2_-ZIF-8 was incorporated into the CTA/CDA membrane.
4. The CO_2_/CH_4_ selectivity can be further increased to 41 under mixed gas tests due to the competitive diffusion and sorption between CO_2_ and CH_4_ molecules.
5. A notable improvement in CO_2_-induced plasticization pressure from 10.4 to 21.47 bar was observed owing to the good compatibility and chain interactions between CTA/CDA and NH_2_-ZIF-8 molecules.

Therefore, the newly fabricated polymer blend MMMs may have great potential for CO_2_ separation in the natural gas industry. 

## Figures and Tables

**Table 1:** Comparison of pure gas separation performance and decomposition temperature (Td) of CTA/CDA-NH_2_-ZIF-8 MMMs with different NH_2_-ZIF-8 loadings.

Sample Name	Actual NH_2_-ZIF-8 Loading (%)	Td (°C)	Pure Gas Permeabilities (Barrer)		CO_2_/CH_4_ Selectivity
			CO_2_	CH_4_	
CTA/CDA- 0 wt.% NH_2_-ZIF-8	0	310	7.56 ± 0.17	0.28 ± 0.02	27.00
CTA/CDA- 5 wt.% NH_2_-ZIF-8	5.13	320	8.10 ± 0.07	0.29 ± 0.03	27.93
CTA/CDA- 10 wt.% NH_2_-ZIF-8	10.58	338	9.52 ± 0.05	0.31 ± 0.01	30.71
CTA/CDA- 15 wt.% NH_2_-ZIF-8	15.80	363	11.33 ± 0.09	0.34 ± 0.02	33.32
CTA/CDA- 20 wt.% NH_2_-ZIF-8	22.37	390	13.20 ± 0.21	0.54 ± 0.01	24.44

**Table 2:** Calculated pure gas permeabilities (Barrer), solubility coefficients (10^−2^ cm^3^ (STP)/cm^3^cm Hg), diffusivity coefficients (10^−8^ cm^2^/s), and corresponding selectivities of CO_2_ and CH_4_ for CTA/CDA-0 wt.% NH_2_-ZIF-8 and CTA/CDA-15 wt.% NH_2_-ZIF-8.

Sample Name	Pure CO_2_			Pure CH_4_			CO_2_/CH_4_ Selectivity		
	P ^a^	S ^b^	D ^c^	P ^a^	S ^b^	D ^c^	$\alpha_{P}$	$\alpha_{S}$	$\alpha_{D}$
CTA/CDA-0 wt.% NH_2_-ZIF-8	7.56	3.47	2.18	0.28	1.29	0.22	27.00	2.69	10.04
CTA/CDA-15 wt.% NH_2_-ZIF-8	11.33	3.61	3.14	0.34	1.35	0.25	33.33	2.67	12.46

^a^ Permeability (Barrer), ^b^ Solubility coefficients (10^−2^ cm^3^ (STP)/cm^3^cm Hg), ^c^ Diffusivity coefficients (10^−8^ cm²/s).

**Table 3:** Comparison of pure gas and mixed gas separation performance.

Sample Name	Pure Gas			Mixed Gas		
	CO_2_ (Barrer)	CH_4_ (Barrer)	Selectivity CO_2_/CH_4_	CO_2_ (Barrer)	CH_4_ (Barrer)	Selectivity CO_2_/CH_4_
CTA/CDA-0 wt.% NH_2_-ZIF-8	7.56	0.28	27	6.70	0.22	30.22
CTA/CDA-15 wt.% NH_2_-ZIF-8	11.33	0.34	33.32	9.50	0.23	41.09

**Table 4:** Comparison of pure-gas separation performance of cellulose acetate (CA)-based membranes in the literature.

Membrane Material	Pres. (Bar)	Temp. (°C )	CO_2_ (Barrer)	CH_4_ (Barrer)	CO_2_/CH_4_	Ref.
CDA	11	35	3.9	0.11	35	[62]
CTA	4	35	6	0.3	20	[49]
CDA-CTA ^a^	11	35	7.1	0.27	26	[34]
CA/nanoporous layered silicate AMH-3	4.6	-	10.36	0.35	30.03	[63]
CA/MWCNTs	2	Room temperature	14.21	0.66	21.20	[64]
P[CA][TF2N] ^b^	1	25	8.9	0.4	22.25	[65]
CTA/[emim][BF_4_] ^c^	4	35	12	0.6	20	[49]
CTA/CDA-0 wt.% NH_2_-ZIF-8	5	35	7.56	0.28	27	This work
CTA/CDA-15 wt.% NH_2_-ZIF-8	5	35	11.33	0.34	33.33	This work

^a^ 20-micron thickness. ^b^ poly(cellulose acetate)(bis(trifluoromethylsulfonyl)imide). ^c^ cellulose triacetate/1-ethyl-3-methylimidazolium tetrafluoroborate([emim][BF4]).

## Data Availability

The data presented in this study are available on request from the corresponding author.
